# Gender and educational attainment influence willingness to donate organs among older Nigerians: a questionnaire survey

**DOI:** 10.11604/pamj.2020.36.288.21125

**Published:** 2020-08-17

**Authors:** Rufus Olusola Akinyemi, Joshua Odunayo Akinyemi, Olorunyomi Felix Olorunsogbon, Ezinne Uvere, Ayodele Samuel Jegede, Oyedunni Sola Arulogun

**Affiliations:** 1 Neuroscience and Ageing Research Unit, Institute for Advanced Medical Research and Training, College of Medicine, University of Ibadan, Ibadan, Nigeria,; 2 Department of Medicine, College of Medicine, University of Ibadan, Ibadan, Nigeria,; 3 Centre for Genomic and Precision Medicine, College of Medicine, University of Ibadan, Ibadan, Nigeria,; 4 Department of Epidemiology and Medical Statistics, College of Medicine, University of Ibadan, Ibadan, Nigeria,; 5 Department of Sociology, Faculty of the Social Sciences, University of Ibadan, Ibadan, Nigeria,; 6 Department of Health Promotion and Education, Faculty of Public Health, University of Ibadan, Ibadan, Nigeria

**Keywords:** Willingness, organ donation, transplantation, Nigeria, Africa, LMIC

## Abstract

**Introduction:**

disparity between the demand for and the supply of organs for transplantation remains a major public health issue of global concern. This study evaluated the knowledge and determinants of willingness to donate organs among outpatient clinic attendees in a Nigerian teaching hospital.

**Methods:**

a 43-item semi-structured interviewer-administered questionnaire was designed to assess awareness and willingness of individuals attending Neurology, Psychiatry and Geriatrics Outpatient clinics to donate bodily organs for transplantation. Association between participants’ characteristics and willingness towards organ donation was investigated using logistic regression models.

**Results:**

a total of 412 participants were interviewed and mean age was 46.3 (16.1) years. There were 229 (55.6%) females and 92.5% had at least 6 years of formal education. Overall, 330 (80.1%) were aware of donation of at least one organ for transplantation purposes but only 139 (33.7%) were willing to donate organ. In analyses, adjusting for sex, marital status, family setting and educational status, male gender AOR [2.066(1.331-3.2016)] secondary education [AOR 5.57 (1.205-25.729) p= 0.028] and post-secondary education [AOR-6.98 (1.537-31.702) p= 0.012 were independently associated with willingness towards organ donation.

**Conclusion:**

the survey revealed high level of awareness but poor willingness towards organ donation among older Nigerians attending outpatient clinics of a premier tertiary hospital. Male gender and educational attainment were significantly associated with willingness to donate. Educational programs that particularly target women and less educated older Nigerians are needed to promote organ donation in Nigeria.

## Introduction

Disparity between organ demand and supply still remains a major public health issue of global concern. The World Health Organization (Geneva) estimates that only 10% of the worldwide need for organ transplantation is being met [1]. Organ shortage is among the greatest challenges in the field of biomedicine today [2]. It has been suggested that if the demands are met, organ replacement could theoretically prevent more than 30% of all deaths in the United States-doubling the average person´s likelihood of living to 80 years of age [3-5]. Epidemiological transition from infectious to non-infectious diseases further increases risk of end stage organ diseases among blacks because chronic conditions such as diabetes and hypertension are particularly common and devastating among individuals of African ancestry [6]. The African continent holds 16% of the world´s population of organ needs but performs only 0.5% of organ transplants [1].

Organ transplantation has raised the life expectancy of end-stage organ diseases patients, offered better quality of life and may be cost effective. For example, the cost of maintenance haemodialysis for one year in Nigeria ranges between US$ 10,000 and US$ 20,000 whereas the cost of kidney transplantation is US$ 20,000 to US$ 30,000 [7]. A number of factors have been reported to influence organ donation rates including educational status of potential donors [8]. Educating potentials donors offers answers to superstitious belief, promotes positive attitudes and increases confidence in becoming a living donor [9]. Decision to donate or not to donate has been reported in many studies as familial. A study done in India reports that family refusal was the commonest reason for unwillingness to be an organ donor [10].

Building a sustainable organ donation program demands a prerequisite understanding of the willingness of potential donors including caregivers to donate organ either for transplantation or research purpose. For instance, a hospital-based study in Ibadan reported that only 26.7% of older clinic outpatients were willing to donate their brain -towards research after death [11]. Exploration of knowledge of and willingness to donate bodily organs will generate relevant information to inform appropriate interventions to improve availability of organs for lifesaving transplantation procedures or mechanistic and discovery laboratory research using post-mortem tissue [12]. This study therefore aimed to explore knowledge and willingness of organ donation and associated factors among older individuals attending the neuroclinics of the University College Hospital, Ibadan south west Nigeria.

## Methods

**Setting**: this study was undertaken in the Neurology and Psychiatry Clinics as well as the Chief Tony Anenih Geriatric Centre (CTAGC) of the University College Hospital Ibadan (UCH), Southwestern Nigeria. UCH is a federal teaching hospital established in I957. It receives referrals from all over Nigeria and is center of excellence in Neurosciences, particularly ageing and dementia research. The CTAGC is the premier purpose-built geriatric centre in Nigeria which caters for individuals 60 years and above, with referrals from across Nigeria.

**Sample**: using a cross-sectional study design, four hundred and twelve consenting adults aged 18-85 years were randomly selected from the Out-patient Clinics of Psychiatry, Geriatrics and Neurology departments of the University College Hospital, Ibadan. A semi-structured questionnaire was used to elicit information from eligible patients and their caregivers after consent has been taken. Trained interviewers interviewed the participants during their waiting time at the various clinics and patients who could not complete the questionnaire before the doctors called them in were monitored to ensure they completed the interview before leaving the hospital during their routine presentation for follow-up. Consenting patients who were adults (>18 years of age); non-demented and mentally stable and capable of participating in discussion were recruited.

**Survey development**: this was a cross sectional study. All data was collected using an interviewer administered semi-structured questionnaire among older adult caregivers and patients visiting the Neuroclinics and Geriatrics centre of the University College Hospital, Ibadan. Further details of the development of the study instrument have been previously described [11].

**Ethical consideration**: all procedures for data collection and analysis for the questionnaire survey phase of the project were reviewed and approved by the University of Ibadan/University College Hospital Institutional Review Board in Ibadan, Nigeria.

**Data collection procedures**: prior to commencement of data collection, a team comprising of eight field staff and volunteers were trained by experts from the Faculty of Public Health of the University of Ibadan on the requisite skills for collecting data using the semi-structured questionnaire and informed consent process. Data collection commenced in the second week of June 2017 and lasted for a period of three weeks. Data was collected by the trained interviewers from qualified and consenting participant by 8 am on Mondays, Tuesdays, Thursdays and Fridays in a week at the Psychiatry, Neurology and Geriatric clinics respectively. Copies of the questionnaire were administered to all consenting participants by trained interviewers and each interview lasted 20 minutes.

**Data analysis procedure**: categorical variables were summarized using frequencies and percentages while mean (SD) was computed for continuous variables. Association between categorical variables and willingness towards brain donation was investigated using the Chi square test. Logistic regression models were fitted to estimate unadjusted and adjusted Odds Ratio (with their 95% Confidence Interval) for factors associated with willingness towards organ donation.

## Results

A total of 412 participants completed the questionnaire survey of which there were 229 females (55.6%). Their mean age was 46.3±16.1 years (Table 1). More than two-fifth of respondents were 50 years and above. About half (50.2%) of the respondents had post-secondary education while few (7.5%) had no formal education. Majority practised Christianity (70.6%) as a religion and were Yoruba (92.7%) by ethnicity. Two hundred and sixty-six (64.6%) were married and mostly in a monogamous setting (79.1%). Table 2 shows the respondents´ knowledge of organs that can be donated. More than four-fifth (80.1%) were aware that kidney can be donated while few considered pancreas (5.6%) and brain (7.5%) as organ that can be donated for transplantation (Table 2). About one-third (33.7%) were willing to donate their organ. Table 2 shows that 25.1% of the female were willing to donate their organ compared with 44.5% of male (p<0.001). Few (6.7%) who had formal education and more than one-third (36.7%) of those who lived in a monogamous family setting were willing to be an organ donor. Other socio-demographic variables including age, religion, ethnic groupings, marital status failed to attain statistical significance (Table 3).

**Table 1:** sociodemographic characteristics of study participants

Variables	mean (SD)
Age(years)	46.3(16.1)
Variables	Frequency (%)
**Age group**	
Below 30	70(17)
30-39	81(19.7)
40-49	83(20.2)
50-59	79(19.2)
60 and above	98(23.9)
**Sex**	
Male	183(44.4)
Female	229(55.6)
**Education**	
No formal education	31(7.5)
Primary	47(11.4)
Secondary	127(30.8)
Post-Secondary	207(50.2)
**Religion**	
Christianity	291(70.6)
Islam and others	121(29.4)
**Ethnic group**	
Yoruba	382(92.7)
Non- Yoruba	30(7.3)
**Marital status**	
Single	107(26.0)
Married	266(64.6)
Widowed/separated/divorced	39(9.4)
**Family setting**	
Monogamous	326(79.1)
Polygamous	86(20.9)

**Table 2:** awareness of organ that can be donated for transplantation (n=412)

Variables	Frequency (%)
Kidney	330 (80.1)
Heart	97(23.5)
Liver	95(23.1)
Cornea	62(15.1)
Bone marrow	53(12.9)
Lung	47(11.4)
Brain	31(7.5)
Pancreas	23(5.6)

**Table 3:** association between socio-demographics and willingness to donate organ

Variables	Willingness to donate organ		p-value
	Yes: 271(33.7%)	No: 138(66.3%)	
**Sex**			
Male	81(44.5)	101(55.5)	<0.001
Female	57(25.1)	170(74.9)	
**Education**			
No formal education	2(6.7)	28(93.3)	0.001
Primary	10(21.3)	37(78.7)	
Secondary	43(34.1)	83(65.9)	
Post-Secondary	83(40.3)	123(59.7)	
**Age**			
Below 30 years	28(20.4)	42(15.5)	0.434
30-39	24(17.5)	56(20.7)	
40-49	32(23.4)	51(18.8)	
50-59	22(16.1)	56(20.7)	
60 and above	31(22.6)	66(24.4)	
**Religion**			
Christianity	97(33.7)	191(66.3)	0.968
Islam and others	41(33.9)	80(66.1)	
**Ethnic group**			
Yoruba	128(33.8)	251(66.2)	0.961
Non-Yoruba	10(33.3)	20(66.7)	
**Marital status**			
Single	43(40.2)	64(59.8)	0.145
Married	86(32.6)	17867.4)	
Widow/separated /divorce	9(23.7)	29(76.3)	
**Family setting**			
Monogamous	119(36.7)	205(63.3)	0.013
Polygamous	19(22.4)	66(77.6)	

Table 4 shows the crude and adjusted odds ratio from a logistic regression model fitted to identify factors that predict respondents´ willingness to be an organ donor. Males were more likely of willing to donate organ than females (AOR=2.10; 95% CI=1.31-3.35). Participants with no formal education were less likely to be willing to donate than those who attained post-secondary school education (AOR=0.14; CI=0.03-0.65). Those who were willing were further asked if they would be willing to inform their family members of their decision to donate organ. Majority (84.8%) were still willing to discuss with their family members about their decision. Analysis of socio-demographic variables and respondents´ willingness to inform family member about consent to donate organ did not show any statistically significant association (Table 5). When asked if they were willing to put their decision to donate in a will. Majority (80.4%) were willing, a slight decline from their willingness to inform family of their willingness to donate. Similarly, there was no relationship between the socio-demographic characteristics and willingness to write organ donation in a will (Table 6). Most (91.3%) of the respondent show willingness to respect family members desire to donate organ after death. However, none of the socio-demographic characteristics demonstrated statistical significance (Table 7).

**Table 4:** factors associated with willingness to donate organ

	Unadjusted			Adjusted		
Variables	OR	95%CI	P Value	OR	95%CI	P Value
Age (years: mean (SD)						
**Age**						
Below 30 years	1					
30-39	0.64	0.33-1.26	0.20			
40-49	0.94	0.49-1.81	0.86			
50-59	0.59	0.30-1.17	0.13			
60-69	0.71	0.37-1.34	0.28			
**Sex**						
Male	2.39	1.57-3.64	**<0.001**	2.07	1.33-3.21	**0.001**
Female	1			1		
						
**Religion**						
Christianity	0.99	0.63-1.55	0.97			
Islam and others	1					
**Ethnicity**						
Yoruba	1.02	0.46-2.24	0.96			
Non-Yoruba	1					
**Marital status**						
Single	1			1		
Married	0.72	0.45-1.14	0.16	0.94	0.57-1.54	0.80
Widowed/separated/divorce	0.46	0.20-1.07	0.07	1.18	0.46—3.02	0.74
**Family setting**						
Monogamous	2.02	1.15-3.52	**0.01**	1.45	0.80-2.64	0.22
Polygamous	1			1		
**Educational status**						
No formal education	1			1		
Primary	3.78	0.77-18.66	0.10	3.30	0.65-16.75	0.15
Secondary	7.25	1.65-31.90	0.01	5.57	1.21-25.73	0.03
Post-secondary	9.45	2.19-40.73	0.003	6.98	1.54-31.70	0.01

Note: only variables with p-value<0.1 from crude OR were included to obtain adjusted ORs using Logit model

**Table 5:** association between socio-demographics and willingness to inform family member about consent to donate organ

Variables	Willingness to inform family member about consent to donate organ		p-value
	Yes: 117(84.8)	No: 21(15.2)	
**Sex**			
Male	66(81.5)	15(18.5)	0.198
Female	51(89.5)	6(10.5)	
**Education**			
No formal education	2(100)	0(0)	0.957*f
Primary	8(80)	2(20)	
Secondary	36(83.7)	7(16.3)	
Post-Secondary	71(85.5)	12(14.5)	
**Age**			
Below 30 years	23(82.1)	5(17.9)	0.855*f
30-39	20(83.3)	4(16.7)	
40-49	28(87.5)	4(12.5)	
50-59	20(90.9)	2(9.1)	
60 and above	25(80.6)	6(19.4)	
**Religion**			
Christianity	82(84.5)	15(15.5)	0.901
Islam and others	35(85.4)	6(14.6)	
**Ethnic group**			
Yoruba	107(83.6)	21(16.4)	0.226*f
Non-Yoruba	10(100)	0(0)	
**Marital status**			
Single	34(79.1)	9(20.9)	0.548*f
Married	75(87.2)	11(12.8)	
Widow/separated /divorce	8(88.9)	1(11.1)	
**Family setting**			
Monogamous	101(84.9)	18(15.1)	1.000*f
Polygamous	16(84.2)	3(15.8)	

**Table 6:** association between socio-demographics and willingness to write organ donation in a will

Variables	Willingness to write organ donation in a will		p-value
	Yes: 111(80.4)	No: 27(19.6)	
**Sex**			
Male	69(85.2)	12(14.8)	0.094
Female	42(73.7)	15(26.3)	
**Education**			
No formal education	1(50)	1(50)	0.640^*^f
Primary	9(90)	1(10)	
Secondary	35(81.4)	8(18.6)	
Post-Secondary	66(79.5)	17(20.5)	
**Age**			
Below 30 years	23(82.1)	5(17.9)	0.435^*^f
30-39	17(70.8)	7(29.2)	
40-49	29(90.6)	3(9.4)	
50-59	17(77.3)	5(22.7)	
60 and above	24(77.4)	7(22.6)	
**Religion**			
Christianity	77(79.4)	20(20.6)	0.631
Islam and others	34(82.9)	7(17.1)	
**Ethnic group**			
Yoruba	101(78.9)	27(21.1)	0.210^*^f
Non-Yoruba	10(100)	0(0)	
**Marital status**			
Single	34(79.1)	9(20.9)	0.942^*^f
Married	70(81.4)	16(18.6)	
Widow/separated /divorce	7(77.8)	2(22.2)	
**Family setting**			
Monogamous	96(80.7)	23(19.3)	1.00^*^f
Polygamous	15(78.9)	4(21.1)	

* f - Fisher´ exact test

**Table 7:** association between socio-demographics and willingness to respect family members desire to donate organ after death

Variables	Willingness to respect family members desire to donate organ after death		p-value
	Yes: 126 (91.3%)	No 12 (8.7%)	
**Sex**			
Male	74(91.4)	7(8.6)	1.000^*^f
Female	52(91.2)	5(8.8)	
**Education**			
No formal education	2(100)	0(0)	0.776^*^f
Primary	10(100)	0(0)	
Secondary	39(90.7)	4(9.3)	
Post-Secondary	75(90.4)	8(9.6)	
**Age**			
Below 30 years	27(96.4)	1(3.6)	0.855^*^f
30-39	21(87.5)	3(12.5)	
40-49	29(90.6)	3(9.4)	
50-59	20(90.9)	2(9.1)	
60 and above	28(90.3)	3(9.7)	
**Religion**			
Christianity	90(92.8)	7(7.2)	0.509^*^f
Islam and others	36(87.8)	5(12.2)	
**Ethnic group**			
Yoruba	116(90.6)	12(9.4)	0.600^*^f
Non-Yoruba	10(100)	0(0)	
Marital status			
Single	40(93)	3(7)	0.569^*^f
Married	77(89.5)	9(10.5)	
Widow/separated /divorce	9(100)	0(0)	
**Family setting**			
Monogamous	107(89.9)	12(10.1)	0.218^*^f
Polygamous	19(100)	0(0)	

* f - Fisher´ exact test

## Discussion

The aim of this study was to assess the knowledge of organ donation and willingness as well as factors that predicts willingness to be an organ donor among outpatient clinic attendees in tertiary hospital in southwestern Nigeria. This is an important step towards understanding strategies to promote organ donation program in our society. Only one third of respondents in this study were willing to donate organs, particularly the male gender and those with higher education. A larger proportion of those willing to be an organ donor considered the need to involve their family members in the decision making process. Educational programs that target family consent should be a good strategy to promote willingness to donate organs in the Nigerian Population. The kidney was the organ respondents were most aware of as a transplantable organ (80.1%), similar to findings among Turkish medical and law students in a previous study [13]. This is also similar to the findings of a study among professional taxi drivers (88.70%) in India [14] and Pakistan where more than 95% were aware of kidney as organ that can be transplanted [15]. This may not be far from the fact that kidney transplantation is increasingly becoming a common procedure in Nigeria.

Willingness to be an organ donor was reported in one- third (33.7%) of our respondents and this was similar to findings in a previous study conducted in Lagos in 2006 where 30% of the respondent were willing to donate organ [16]. A similar proportion (29.5%) was previously reported among health workers in southwestern Nigeria [17]. However, higher proportions (62.3%) have been reported in a Pakistani study [15] and an Indian (81.2%) [18]. Programs geared toward increasing willingness to donate organ have been developed in many countries. Training of health professionals on how to effectively communicate with the family is one approach that has been adopted in UK, Spain and Australia to promote organ donation [13,19,20]. A study reported that the consent rate for organ donation increased from 46.3% to 55.5% after procurement coordinators were trained on effective communication with the family [21] and proper management of the consent process by nurses promoted family likelihood of accepting organ donation [22]. In multivariable logistic regression, male gender and higher educational attainment were associated with organ donation. Participants with no formal education were less likely to be willing to donate their organ. This study also found an association between male gender and willingness to donate organ and this was similarly reported in a study on brain donation in Ibadan [11]. This may be due to African culture that does not give freedom to women to easily take decision on their own [23]. This was in contrast to a study done among health workers in south west, Nigeria which report that a greater percentage of female were willing to be organ donor compared to their male counterpart [17] this difference observed may be due to study settings which has majority (88.6%) of it respondent as female. Hispanic American study also show that more women were more likely to consent to organ donation compare to men [24].

Our study reveals that respondents with no formal education were less likely to show willingness to donate their organs. More than half (52%) of respondents in an Indian study reported that lack of awareness has a negative influence on organ donation [18]. Education tailored towards the benefit of organ donation could reverse this finding, this has been used to promote donation of organ among the African Americans [21]. Backing organ donation decision through a will was welcomed by a majority (80.4%) in this study. This could be seen as opt-in method, that is, a potential donor voluntarily agrees to donate although family agreement with consent for organ donation by the deceased has been reported in studies as a key determinant of organ donation practice [25]. This was not significant but majority in our study, irrespective of gender, age, religion, marital status, educational status and family settings shows that they will be willing to inform family members of their decisions to be an organ donor. This may pose some barriers to organ donation and may reduce the numbers of those who actualise their willingness to be an organ donor. Culture-specific information should be designed to address family views. Consent rate for organ donation will be promoted if family, language, culture, faith and values are considered in the donation process [26]. These are worthy of deeper exploration particularly using qualitative approaches.

## Conclusion

Willingness to donate organ was reported in about one-third of respondents in our study. It was found that educational status and being a male gender promote willingness to donate organs. Culturally appropriate educational programs that target the female gender and less educated individuals will be helpful in promoting organ donation in Nigeria.

### What is known about this topic

- Demand for organs for transplantation far outstrips the supply;
- Educational status is a key factor influencing decisions to donate organs.

### What this study adds

- The facilitating influence of the male gender for decision in favour of organ donation in southwestern Nigeria;
- Readiness to express willingness to be an organ donor in a written will in a cohort of older adult Nigerians;
- Expression of willingness to respect family members´ decision to donate their organs after death.

## References

[1] Jones B, Bes M (2012). Keeping kidneys. Bull World Health Organ.

[2] Giwa S, Lewis JK, Alvarez L, Langer R, Roth AE, Church GM (2017). The promise of organ and tissue preservation to transform medicine. Nature biotechnology.

[3] Fahy GM, Wowk B, Wu J (2006). Cryopreservation of complex systems: the missing link in the regenerative medicine supply chain. Rejuvenation research.

[4] Lefaucheur C, Glotz D (2014). Desensitization protocols for organ transplantation. Wiley Online Library.

[5] Peters TG (1991). Life or death: the issue of payment in cadaveric organ donation. JAMA.

[6] Roderick PJ, Raleigh VS, Hallam L, Mallick NP (1996). The need and demand for renal replacement therapy in ethnic minorities in England. Journal of Epidemiology & Community Health.

[7] Shonibare A The transplantation experience in the Sub-Saharan region SNH series: in conference paper presentation. Primier Conference Africane sur la Transplantation des Organs. Bamako meeting.

[8] Abbasi M, Kiani M, Ahmadi M, Salehi B (2018). Knowledge and ethical issues in organ transplantation and organ donation: perspectives from iranian health personnel. Ann Transplant.

[9] Mithra P, Ravindra P, Unnikrishnan B, Rekha T, Kanchan T, Kumar N (2013). Perceptions and attitudes towards organ donation among people seeking healthcare in tertiary care centers of coastal South India. Indian journal of palliative care.

[10] Balajee KL, Ramachandran N, Subitha L (2016). Awareness and attitudes toward organ donation in rural Puducherry, India. Annals of medical and health sciences research.

[11] Rufus Akinyemi, Akin Ojagbemi, Joshua Akinyemi, Ayodeji Salami, Funmi Olopade, Temitope Farombi (2019). Gender differential in inclination to donate brain for research among Nigerians: the IBADAN Brain Bank Project. Cell Tissue Bank.

[12] Akinyemi RO, Salami A, Akinyemi J, Ojagbemi A, Olopade F, Coker M (2019). Brain banking in low and middle-income countries: raison d'être for the Ibadan brain ageing, dementia and neurodegeneration (IBADAN) brain bank project. Brain Res Bull.

[13] Consent/Authorisation Best Practice Development Group (2015). Approaching the families of potential organ donors: best practice guidance. NHS Blood and Transplant, UK.

[14] Jagadeesh AT, Puttur A, Mondal S, Ibrahim S, Udupi A, Prasanna LC (2018). Devising focused strategies to improve organ donor registrations: a cross-sectional study among professional drivers in coastal South India. PloS one.

[15] Saleem T, Ishaque S, Habib N, Hussain SS, Jawed A, Khan AA (2009). Knowledge, attitudes and practices survey on organ donation among a selected adult population of Pakistan. BMC medical ethics.

[16] Odusanya OO, Ladipo CO (2006). Organ donation: knowledge, attitudes, and practice in Lagos, Nigeria. Artificial organs.

[17] Oluyombo R, Fawale MB, Ojewola RW, Busari OA, Ogunmola OJ (2016). Knowledge regarding organ donation and willingness to donate among health workers in South-West Nigeria. International journal of organ transplantation medicine.

[18] Panwar R, Pal S, Dash NR, Sahni P, Vij A, Misra MC (2016). Why are we poor organ donors: a survey focusing on attitudes of the lay public from northern India. Journal of clinical and experimental hepatology.

[19] Organ and Tissue Authority The Australasian Donor Awareness Program (ADAPT).

[20] Vincent A, Logan L (2012). Consent for organ donation. British journal of anesthesia.

[21] Siminoff LA, Marshall HM, Dumenci L, Bowen G, Swaminathan A, Gordon N (2009). Communicating effectively about donation: an educational intervention to increase consent to donation. Progress in Transplantation.

[22] Cebeci F, Sucu G, Karazeybek E (2011). The roles of nurses to augment organ donation and transplantation: a survey of nursing students. InTransplantation proceedings.

[23] Dodoo FN (1998). Marriage type and reproductive decisions: a comparative study in sub-Saharan Africa. Journal of Marriage and the Family.

[24] Alvaro EM, Jones SP, Robles AS, Siegel JT (2005). Predictors of organ donation behavior among Hispanic Americans. Progress in Transplantation.

[25] Julius Weiss, Michael Coslovsky, Isabelle Keel, Franz Immer, Peter Jüni (2014). Comit&eacut; National du Don d´Organes. Organ donation in Switzerland-an analysis of factors associated with consent rate. PLoS One.

[26] Goldberg DS, French B, Abt PL, Gilroy RK (2015). Increasing the number of organ transplants in the United States by optimizing donor authorization rates. American Journal of Transplantation.

